# Stress and recovery perception, creatine kinase levels, and performance parameters of male volleyball athletes in a preseason for a championship

**DOI:** 10.1186/s40798-020-00255-w

**Published:** 2020-06-26

**Authors:** Guilherme Pereira Berriel, Rochelle Rocha Costa, Edson Soares da Silva, Pedro Schons, Guilherme Droescher de Vargas, Leonardo Alexandre Peyré-Tartaruga, Luiz Fernando Martins Kruel

**Affiliations:** Exercise Research Laboratory, School of Physical Education, Physical Therapy and Dance, Universidade Federal do Rio Grande do Sul, 750 Felizardo Street, Porto Alegre, Rio Grande do Sul 90690-200 Brazil

**Keywords:** Physical performance, Vertical jump, Training load, Overtraining, Psychological markers, Muscular enzymes

## Abstract

**Background:**

Training load and adequate recovery have been identified as essential elements to improve well-being and performance in team sports and avoid non-functional overreaching and overtraining.

**Objective:**

This cohort study sought to analyze the stress and recovery perceptions, creatine kinase levels (CK), and vertical jump performance of volleyball athletes at different training times during a championship preseason.

**Methods:**

Thirteen high-level male volleyball players (23.80 ± 5.40 years, 91.50 ± 8.80 kg, and 193.10 ± 6.40 cm) completed the RESTQ-Sport questionnaire on stress and recovery perception, and blood samples were collected to evaluate CK levels. These measures were performed six times over 16 weeks, while jumps such as squat jump (SJ), countermovement jump (CMJ), and countermovement jump arm (CMJA) were performed at three of those times for specific performance evaluation.

**Results:**

The stress perception and recovery perception indices increased and decreased, respectively, in the pre-competitive phase, while CK levels presented an initial rise, maintenance over the training period, and a drop. Vertical jump heights increased significantly throughout the preparatory period regardless of the type of jump. In all training phases, CMJA values exceeded CMJ and SJ values, and CMJ values exceeded SJ values.

**Conclusions:**

Positive adaptations were elicited by training stimuli, resulting in improvements in performance. Conversely, load training variables indicated higher levels of stress and muscle damage, together with lower perceptions of recovery during the championship preseason.

## Key points

- The perceptions of both stress and recovery tended to remain stable during the preparatory phase, and the perception of stress increased and that of improvement decreased close to competition.
- Muscle damage increased soon after the first few weeks of training start and tended to remain stable until the pre-competitive phase, at which time levels significantly decreased.
- Although the variables used to monitor training indicated higher levels of stress and muscle damage, in addition to lower perceptions of recovery, positive adaptations were made to the training stimuli, with resulting improvements in athletes’ performance.

## Introduction

Team sports improve physical performance and enhance social connections. Concerning physical performance, training methods play an essential role in preserving physical integrity and maximizing the athlete’s performance [1]. In this regard, it is necessary to establish a specific workload to ensure the best performance [2]. In the course of the training process, there is a constant imbalance of homeostasis provided by the training load, which is a source of psychophysiological and biomechanical stresses fundamental to athletic performance [3–6]. Athletes are often subjected to high training loads of long duration, high intensity, and elevated frequency of the stimulus. These parameters are called “external loads”, while in contrast, the physiological and psychological stresses imposed on the athlete can be called “internal load” [7].

A common strategy in high-performance sports to improve physical fitness is to alternate periods of high and low training loads [6]. After a period of high training loads, there may be a decline in performance [8] which, with proper recovery, returns to previous levels or even improves [9, 10]. During a period of intense training, it is expected that the athlete will improve their performance, that is, adapt to the stress to which he was subjected. However, some athletes may show a decline in performance, which has been attributed to specific psychological and physiological processes [6, 11]. Verkoshanky [12] points out that high training loads can endanger the athlete’s health. In this context, a common phenomenon in the sports environment is overtraining. Excess training, lack of adequate recovery, and other stressors within or outside the training context result in some physiological changes and may result in a decline in athlete performance [13]. From a physiological point of view, it has been demonstrated that a substantial increase of creatine kinase enzyme (CK) levels combined with reduced exercise tolerance is a critical overtraining marker [14]. Additionally, it demonstrated exacerbated CK levels when compared to male athletes’ and healthy sedentary controls’ levels [15]. In 2013, the American College of Sports Medicine [16] suggested that the best treatment for overtraining would be to withdraw from sports activities and rest for a period of a few weeks or even months. Thus, it would be advantageous to prevent the development of overtraining syndrome [16]. The clinical manifestation of overtraining occurs through a series of signs and symptoms representative of physiological, psychological, and behavioral aspects of performance and performance-related changes [13].

Some studies have investigated the effects of periods of training of athletes in preparation for professional championships [17–19], as well as physiological [1, 8, 17] and performance [20, 21] parameters in isolation. However, no study was found in the literature that presented the findings for all these parameters together in the same athletes, or even in athletes of the same sport. Such results could provide valuable information for a complete analysis of the team’s responsiveness to the proposed protocols during preseason and allow inferences about probable interventions.

The recent achievements of men’s and women’s volleyball teams have increased the popularity and promoted the development of this sport, which has come to be treated with greater professionalism due to its growing competitiveness [22]. Given the practical realities of current volleyball, the need has arisen to better control the training variables by identifying and characterizing the competitive level of the athletes and adapting the conditioning and requirements of physical and physiological parameters accordingly. Therefore, the present study aims to analyze the stress perception, recovery perception, creatine kinase levels, and performance in jump tests of volleyball athletes in different periods of training during a volleyball championship preseason. We hypothesize that stress perception, recovery perception, and creatine kinase levels will oscillate with changes in training loads. In addition, we hypothesize that the performance in jump tests will increase over the period assessed.

## Methods

### Participants

The subjects of this study, 13 male athletes from a Brazilian volleyball team, were recruited by convenience. This volleyball team participates in the First Division Volleyball National League (*Superliga*). We adopted as exclusion criteria previous injuries within the last 6 months, which limits training periodization and performance of physical capacities. From a hormonal point of view, participants were not in current or recent use of hormones, use of glucocorticoids, or use of centrally acting drugs; did not have current medical conditions that may affect perceptions; and were not in regular use of muscle-relaxing drugs. Also, participants should have at least 2 years of experience in national and international competitions and have been part of the Brazilian National Team. The Ethics Committee of the Federal University of Rio Grande do Sul approved the study (No. 1.464.312), which was conducted in accordance with the Declaration of Helsinki. All participants were informed regarding the procedures of the study and its risks and benefits, and informed consent was then obtained from the individual participants.

### Study design

We used the Strobe checklist for this study. It was a cohort study. The training sessions of the volleyball team were monitored by a rate of perceived exertion (RPE) scale and perceived recovery status (PRS) during a volleyball championship preseason. The preseason was divided into a preparatory and a pre-competitive period. The volleyball team competed in a regional championship in the pre-competitive period. The technical committee organized the training periodization for a period of 16 weeks (Table 1). The athletes returned from an off-season period during which physical activities were not controlled, and all subjects performed a set of tests at seven moments: The first moment (M1) was performed at the beginning of the session, the second moment (M2) 1 week after M1, the third moment (M3) 3 weeks after M2, the fourth moment (M4) 2 weeks after M3, the fifth moment (M5) 2 weeks after M4, the sixth moment (M6) 3 weeks after M5, and the seventh moment (M7) 4 weeks after M6. Vertical jump performance was evaluated in the afternoon at only M1, M4, and M7, and at M7 only vertical jumps were evaluated. This assessment was scheduled then because of the proximity of the competition. The difference between the weeks in the moments of the tests was due to the team’s availability and schedule. The application of the tests always followed the same sequence, starting with a standard warm-up and using the following order for the tests: 1st, squat jump (SJ); 2nd, countermovement jump (CMJ); and 3rd, countermovement jump arm (CMJA). The blood samples were always collected in the morning after a day and a half of rest and before the first training activity of the week. The Recovery-Stress Questionnaire for Athletes (RESTQ-Sport) was applied the morning before starting the daily training sessions. These measures were performed at moments 1 to 6. These dates were suggested for evaluation as substantial changes in the training load had been programmed by the technical committee during these periods. For the control of the training load, 100% of the weekly load was considered a situation in which training was carried out every day of the week with two shifts per day, being 80% from week 1 to 4, 85% from week 5 to 8, 90% from week 9 to 12, and 80% from week 13 to 16. The quantification of the training load was performed by counting the time, in minutes (Table 1), without considering the recovery and rest time.

**Table 1:** Description of the general characteristics and training load at each evaluation moment

Weeks	Week 1	Week 2	Week 3	Week 4	Week 5	Week 6	Week 7	Week 8	Week 9	Week 10	Week 11	Week 12	Week 13	Week 14	Week 15	Week 16
Period	PP	PP			PP		PP		PC			PC				
Goal	P	MS-TT			MS-TT		ES-TT		ES-TT			V-TT				
Number of Games							4		4			5				
Total Weekly Training Load (min)	879				1153		1325		1055			1105				
Moment of Evaluation	M1	M2			M3		M4		M5			M6				M7
Blood Sample Assessment	x	x			x		x		x			x				
RESTQ-Sport Assessment	x	x			x		x		x			x				
Vertical Jump Assessment	x						x									x

*M1* first moment of evaluation, *M2* second moment of evaluation, *M3* third moment of evaluation, *M4* fourth moment of evaluation, *M5* fifth moment of evaluation, *M6* sixth moment of evaluation, *P* presentation, *MS* muscular strength, *TT* tactical-technician, *ES* explosive strength, *V* velocity, *PP* preparatory period, *PC* pre-competitive period

## Procedure

### Psychological variables

RESTQ-Sport [23], which was used to assess stress and recovery status, was completed by the athletes in a meeting room, where their privacy and tranquility could be ensured. In addition, the athletes were informed that technicians would not have access to the questionnaire results during the session. For data analysis, we used the RESTQ-Sport® program on Windows® to calculate mean values for each of these 19 items: (1) general stress, (2) emotional stress, (3) social stress, (4) conflicts/pressure, (5) fatigue, (6) energy loss, (7) physical complaints, (8) success, (9) social recovery, (10) physical recovery, (11) general well-being, (12) sleep quality, (13) interval disturbances, (14) emotional exhaustion, (15) injuries, (16) fitness, (17) personal acceptance, (18) self-efficacy, and (19) self-regulation.

### Creatine kinase (CK)

We collected fingertip capillary samples to determine the CK concentration by reflectance photometry at 37 °C using a calorimetric assay procedure (Reflotron, Boehringer Mannheim, Germany) [24]. The calibration of the apparatus followed the standard recommendations. After finger sterilization with alcohol, a blood sample of 30 μl was collected by digital puncture and was immediately placed in specific reagent strips inserted into the apparatus.

### Vertical jump performance

To examine the variability of physical capacities (jump height) during the session, we evaluated SJ, CMJ, and CMJA performance with a contact mat (Jump test®, Hidrofit Ltda, Brazil). The participants performed a standard warm-up of 10 min of running and jumping at submaximal intensity before the beginning of the test. For SJ, the participants completed a maximal vertical jump with their hands on the hips from 90° flexed knee position without countermovement. For CMJ, the athletes started from a standing position with their hands on the hips, and then they performed a maximal vertical jump with a quick flexion of hip and knee (approximately 90°) followed by the total extension with countermovement [25]; the test for CMJA was similar to that of CMJ except that the athletes used their hands to boost their body [26]. The CMJA was chosen because it approximates the volleyball context. We chose the best performance out of three trials for all vertical jumps (SJ, CMJ, CMJA).

### Statistical analysis

Data are presented as means and standard deviations. Normality was tested using the Shapiro-Wilk test. The Friedman test was adopted to verify possible differences in the CK levels and psychometric variables at different time points (M1 to M6). For those cases where significant differences were found, the Wilcoxon test was used to identify the significantly different moments. Additionally, the generalized estimating equations were used to verify the differences between the heights of the jumps (adopting the “type of jump” and the “moment” as factors). The Bonferroni post hoc test was used to locate the differences. Finally, secondary analyses were performed, aiming to verify whether there are associations between the stress and recovery perceptions, creatine kinase levels, and the performance parameters, using Pearson’s and Spearman’s correlation coefficients. For all analyses, the significance level was set at *α* ≤ 0.05 and SPSS (Statistical Package for the Social Sciences for Mac, version 22.0, IBM, USA) was used.

## Results

The results of the present study were obtained from 13 volleyball athletes with a mean age of 23.80 ± 5.40 years, mean weight of 91.50 ± 8.80 kg, and mean height of 193.10 ± 6.40 cm. All athletes participated in all evaluations.

During the preseason preparatory period when the data of this study were collected, the average weekly training volume increased from 879 min between M1 and M2 to 1153 min between M2 and M3, and to 1325 min between M3 and M4 (Table 1). During the period corresponding to M2 and M3, the training sessions comprised muscle strength training which consisted of two exercises for each muscular group with 80 to 90% of 1RM four times for the week added to technical-tactical exercises, and there were no competitive games during this period. The exercises performed for upper limbs were bench press, flat dumbbell fly, alternate shoulder press, barbell shoulder press, Scott biceps, unilateral biceps, French triceps, unilateral cable triceps, rowing bar, and lat pulldown machine. For the lower limbs, the following exercises were used: squat, deadlift, knee extension machine, calf stand up, calf sit down, knee flexor machine, adduction machine, and abduction machine. During the period corresponding to M4 and M5, the muscular strength training emphasized explosive strength which was based on plyometric training that consisted of jump exercises and technical-tactical exercises were maintained. Between M4 and M5, the team participated in four games (Table 1).

In the pre-competitive period, the training lasted 1055 min (weekly average) between M4 and M5, and 1105 min (weekly average) between M5 and M6, and between M4 and M5 and M5 and M6, respectively, the team participated in four and five games which were not included in the training load. During the period between M5 and M6, explosive strength training was replaced by velocity training based on plyometric training consisting of drop jumps ranging in 30 to 50 cm twice a week and a volume of 90 jumps without overload, while technical-tactical training was maintained (Table 1).

Results of general stress, sport-specific stress, general recuperation, sport-specific recuperation, CK levels, and vertical jumps are presented in Table 2.

**Table 2:** Description of the main results

Variables	M1	M2	M3	M4	M5	M6	M7
**General stress**							
General stress	0.23 ± 0.39	0.19 ± 0.37	0.21 ± 0.42	0.48 ± 0.59	0.50 ± 0.74	0.38 ± 0.63	
Emotional stress	0.52 ± 0.43	0.60 ± 0.61	0.52 ± 0.43	0.73 ± 0.69	1.79 ± 0.71	1.80 ± 0.66	
Social stress	0.23 ± 0.38	0.29 ± 0.46	0.29 ± 0.40	0.56 ± 0.59	2.15 ± 0.71	2.13 ± 0.75	
Conflicts/pressure	1.50 ± 0.81	1.38 ± 0.98	1.38 ± 0.80	1.46 ± 0.83	2.92 ± 0.62	3.00 ± 0.63	
Fatigue	0.29 ± 0.33	1.50 ± 0.98	0.40 ± 0.36	1.75 ± 0.73	2.67 ± 0.56	2.79 ± 0.50	
Lack of energy	0.54 ± 0.49	0.48 ± 0.49	0.46 ± 0.40	0.81 ± 0.55	3.27 ± 0.69	3.13 ± 0.52	
**Sport-specific stress**							
Disturbed breaks	0.60 ± 0.90	0.77 ± 0.65	0.79 ± 0.77	1.00 ± 0.67	2.27 ± 0.82	2.14 ± 0.57	
Emotional exhaustion	0.42 ± 0.57	0.46 ± 0.54	0.40 ± 0.49	0.52 ± 0.41	1.98 ± 0.73	1.98 ± 0.58	
Injury	1.13 ± 1.19	2.48 ± 1.01	1.81 ± 0.95	2.69 ± 0.97	2.85 ± 0.67	2.64 ± 0.62	
**General recuperation**							
Success	3.56 ± 1.05	4.23 ± 0.86	3.88 ± 0.75	4.15 ± 1.03	2.56 ± 0.33	2.45 ± 0.49	
Social recovery	4.21 ± 0.90	4.40 ± 0.79	4.29 ± 0.86	4.17 ± 0.88	3.10 ± 0.69	2.80 ± 0.75	
Physical recovery	4.54 ± 1.28	3.63 ± 1.13	4.19 ± 1.11	3.37 ± 0.79	1.52 ± 0.53	1.55 ± 0.48	
General well-being	5.08 ± 0.65	5.08 ± 0.75	5.00 ± 0.63	4.90 ± 0.86	3.06 ± 0.90	2.91 ± 0.60	
Sleep quality	5.21 ± 0.59	4.65 ± 0.94	5.08 ± 0.40	4.61 ± 0.67	3.67 ± 0.92	3.55 ± 0.90	
**Sport-specific recuperation**							
Being in shape	4.85 ± 0.89	4.25 ± 0.99	4.71 ± 0.79	4.33 ± 0.45	1.63 ± 0.55	1.50 ± 0.67	
Personal accomplishment	4.65 ± 0.86	4.71 ± 0.93	4.69 ± 0.91	4.65 ± 0.97	2.71 ± 0.52	2.48 ± 0.62	
Self-efficacy	4.85 ± 0.69	4.71 ± 0.77	4.71 ± 0.68	4.25 ± 0.92	3.75 ± 0.51	3.66 ± 0.52	
Self-regulation	4.98 ± 0.82	4.94 ± 1.02	4.98 ± 0.86	5.21 ± 0.86	0.92 ± 1.11	0.82 ± 0.75	
**Creatine kinase (CK)**							
CK levels	169.01 ± 94.42	673.92 ± 461.45	520.77 ± 348.87	631.76 ± 579.30	270.78 ± 245.37	330.23 ± 206.98	
**Vertical jump height**							
Squat jump	35.8 ± 5.0			39.7 ± 5.5			42.9 ± 6.2
Countermovement jump	40.0 ± 4.6			44.4 ± 5.2			51.6 ± 8.9
Countermovement jump arm	46.7 ± 6.1			51.6 ± 8.9			55.6 ± 9.7

*M1* first moment of evaluation, *M2* second moment of evaluation, *M3* third moment of evaluation, *M4* fourth moment of evaluation, *M5* fifth moment of evaluation, *M6* sixth moment of evaluation, *M7* seventh moment of evaluation

**Fig. 1:**
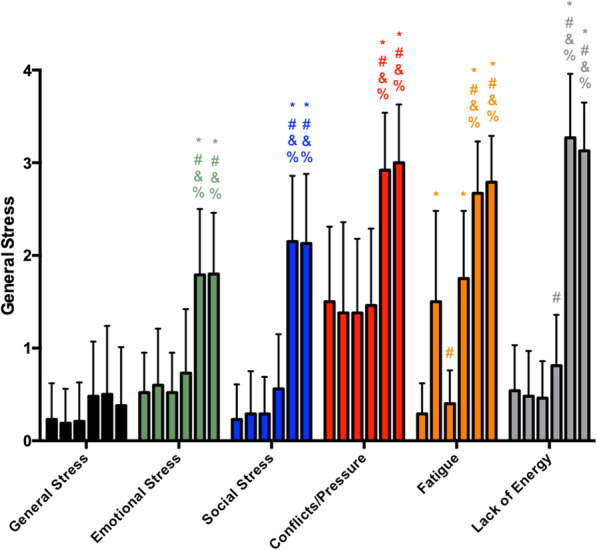
General stress scores during the preseason (each vertical bar, of each parameter, corresponds to a moment of analysis, in order, from moment 1 to moment 6). The asterisk indicates significant difference to M1, number sign indicates significant difference to M2, ampersand indicates significant difference to M3, and percent sign indicates significant difference to M4

Regarding the results for general stress (Fig. 1), it was observed that the scores were maintained at much the same level throughout the whole preseason, while emotional stress, social stress, and conflicts/pressure increased at M5 and M6 over the previous moments. Fatigue perception presented a significant increase from M1 to M2, a decrease from M2 to M3, maintenance from M3 to M4, and another increase from M4 to M5, followed by maintenance from M5 to M6, while lack of energy remained unchanged from M1 to M3, increased at M4 relative to M2, and showed a considerable increase atM5 and M6 (without a significant difference between the M5 and M6 scores). In summary, the scores of all the scales except general stress were highest during the entire preseason at M5 and M6.

**Fig. 2:**
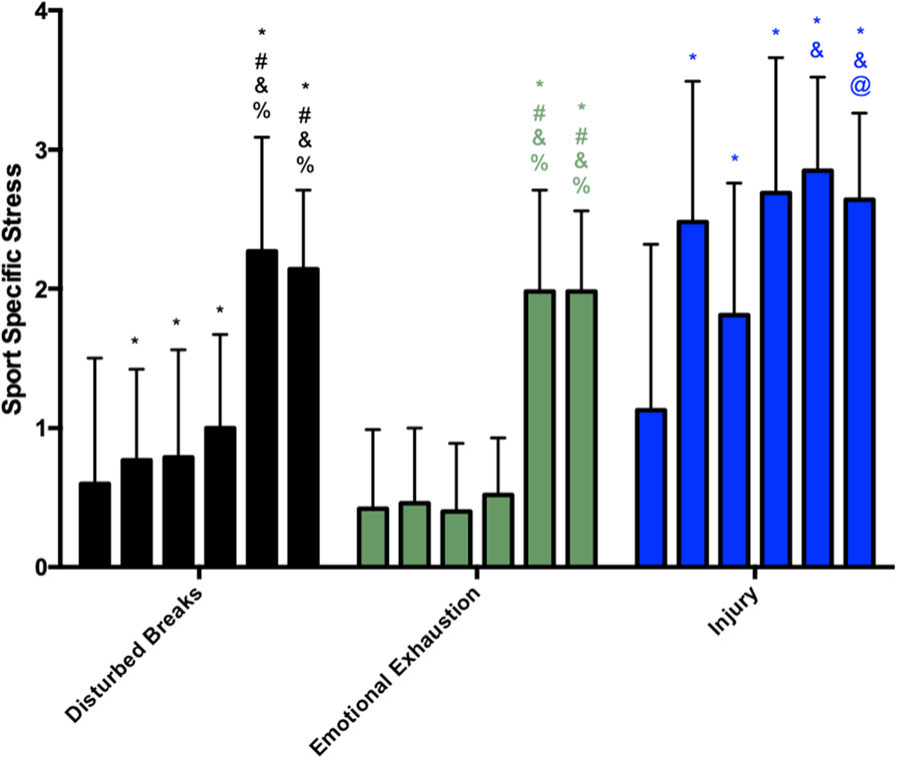
Sport-specific stress scores during the preseason (each vertical bar, of each parameter, corresponds to a moment of analysis, in order, from moment 1 to moment 6). The asterisk indicates significant difference to M1, number sign indicates significant difference to M2, ampersand indicates significant difference to M3, and percent sign indicates significant difference to M4

Similarly, the scores of sport-specific stress presented increases during the preseason (Fig. 2). Disturbed breaks increased from M1 to M2 and then remained unchanged until M4, then showed a rise at M5 and M6 (with no differences between M5 and M6). Emotion exhaustion remained unchanged from M1 to M4 and increased at M5 and M6 (without a difference between them). The injury scores showed a significant increase from M1 to M2 and then remained unchanged until M4, whereupon they presented a rise at M5 (relative to M3) and a decrease at M6 relative to M5. In summary, the scores of all the scales were the highest at M5 and M6 throughout the preseason.

**Fig. 3:**
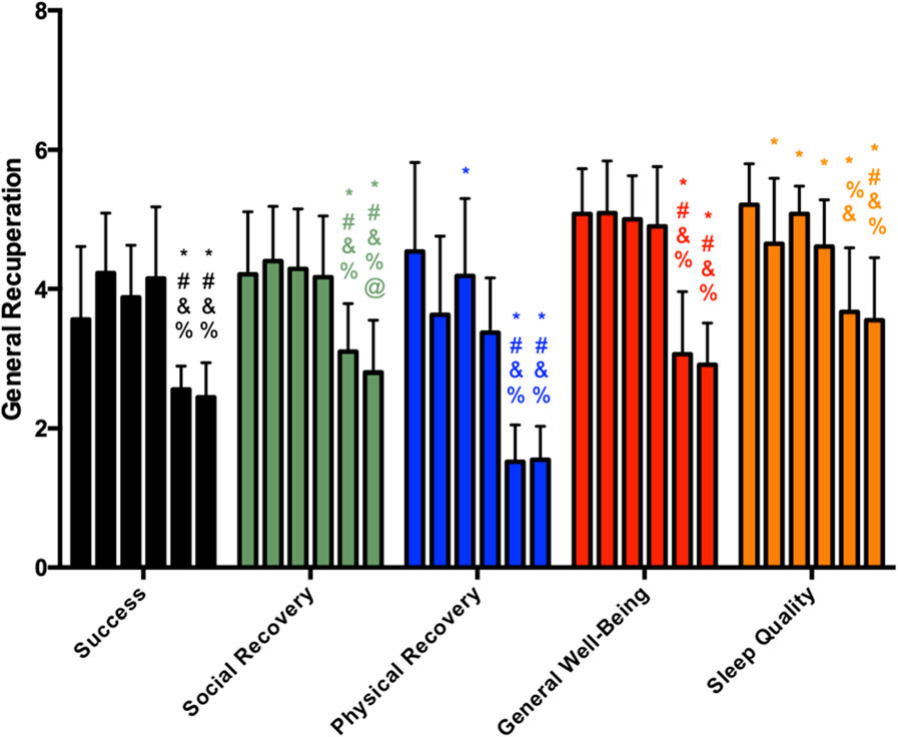
General recuperation scores during the preseason (each vertical bar, of each parameter, corresponds to a moment of analysis, in order, from moment 1 to moment 6). The asterisk indicates significant difference to M1, number sign indicates significant difference to M2, ampersand indicates significant difference to M3, and percent sign indicates significant difference to M4

The scores of general recuperation during the preseason are presented in Fig. 3. Success, social recovery, and general well-being remained unchanged until M4 and then presented decreases in M5, with these lower values maintained at M6 except for social recovery, whose scores decreased even more at M6. Physical recovery decreased at M3 (in relation to M1), remained unchanged at M4, and dropped at M5 and M6 (without a difference between M5 and M6). Sleep quality scores reduced from M1 to M2, remained unchanged until M4, and presented a decrease at M5, with these lower values being maintained at M6. In summary, the scores of all these scales were the lowest at M5 and M6 throughout all the preseason.

**Fig. 4:**
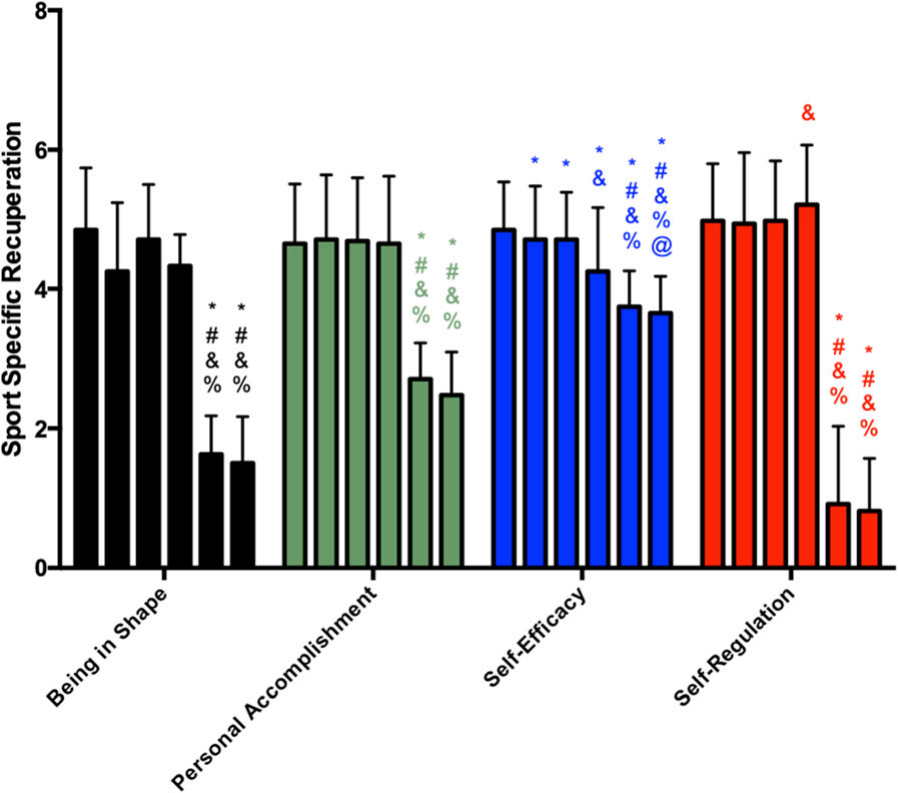
Sport-specific recuperation scores during the preseason (each vertical bar, of each parameter, corresponds to a moment of analysis, in order, from moment 1 to moment 6). The asterisk indicates significant difference to M1, number sign indicates significant difference to M2, ampersand indicates significant difference to M3, and percent sign indicates significant difference to M4

The scores for sport-specific recuperation are presented in Fig. 4. Being in shape and personal accomplishment scales showed maintained scores from M1 to M4 and a significant drop at M5, with these lower values being maintained at M6. Self-efficacy showed a reduction from M1 to M2 and maintenance to M3, and further decreases were observed at M4, M5, and M6. Self-regulation maintained its scores from M1 to M3, then saw a significant increase at M4, and finally similar values at M5 and M6. In summary, the scores of all the scales were the lowest at M5 and M6 during the preseason.

**Fig. 5:**
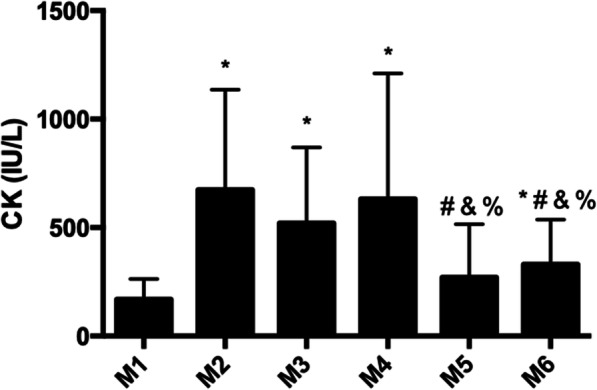
Creatine kinase (CK) concentrations during the preseason (M1, first moment of evaluation; M2, second moment of evaluation; M3, third moment of evaluation; M4, fourth moment of evaluation; M5, fifth moment of evaluation; M6, sixth moment of evaluation). The asterisk indicates significant difference to M1, number sign indicates significant difference to M2, ampersand indicates significant difference to M3, and percent sign indicates significant difference to M4

The CK levels measured at the six moments of the preseason are presented in Fig. 5. CK levels increased by 504.91 IU/L from M1 to M2 (*p* = 0.001) and remained unchanged from M2 to M3 (*p* = 0.249), and from M3 to M4 (*p* = 0.311). A decrease of 360.98 IU/L was observed from M4 to M5 (*p* = 0.004), and maintenance in the values of CK was obtained from M5 to M6 (*p* = 0.084).

**Fig. 6:**
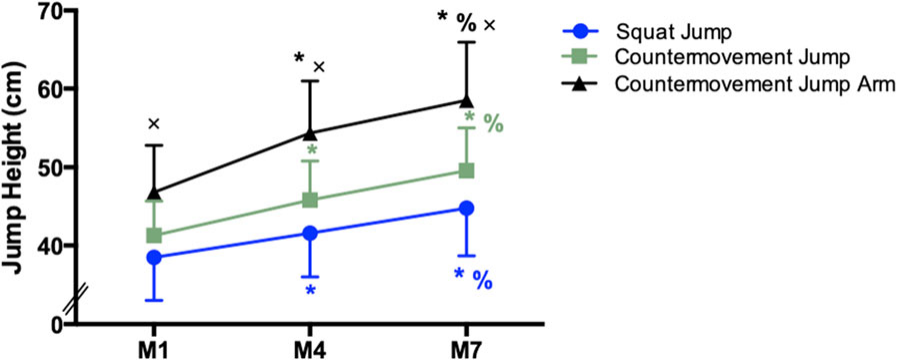
Jump heights during the preseason (M1, first moment of evaluation; M4, fourth moment of evaluation; M7, seventh moment of evaluation). The asterisk indicates significant difference to M1, percent sign indicates significant difference to M4, and “x” indicates significant difference between the three jumps at the same moment

Jump heights increased during the preseason (Fig. 6). The increases from M1 to M4 were 3.12 cm in the SJ (*p* = 0.002), 4.52 cm in the CMJ (*p* < 0.001), and 7.55 cm in the CMJA (*p* < 0.001). Similarly, improvements were observed from M4 to M7 of 3.21 cm in the SJ (*p* < 0.001), 3.79 cm in the CMJ (*p* < 0.001), and 4.20 cm in the CMJA (*p* < 0.001). Furthermore, the mean heights of the three types of jumps were different at M1 (SJ × CMJ: *p* = 0.001; SJ × CMJA: *p* < 0.001; CMJ × CMJA: *p* < 0.001), at M4 (SJ × CMJ: *p* < 0.001; SJ × CMJA: *p* < 0.001; CMJ × CMJA: *p* < 0.001), and at M7 (SJ × CMJ: *p* < 0.001; SJ × CMJA: *p* < 0.001; CMJ × CMJA: *p* < 0.001).

**Fig. 7:**
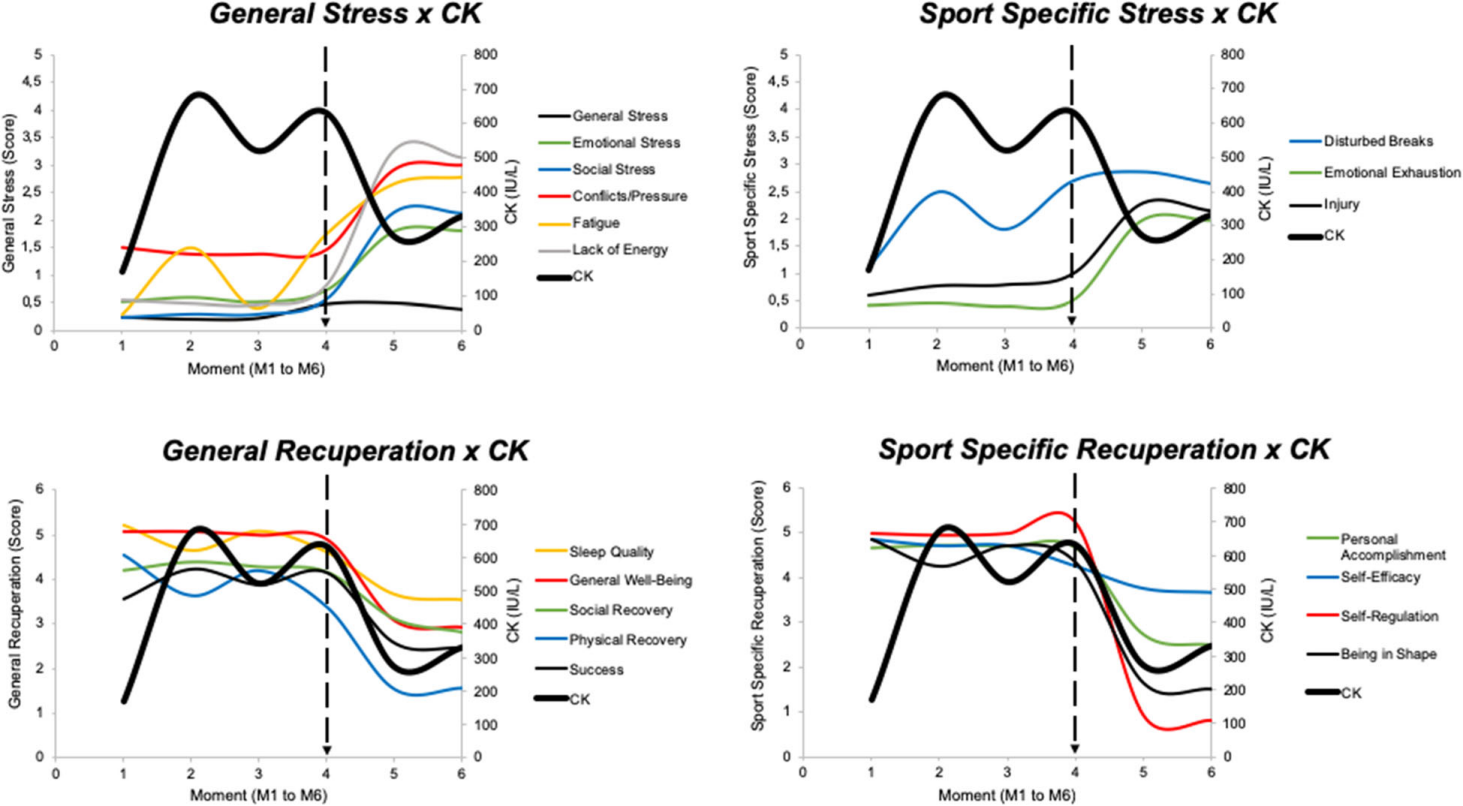
General view of the variables’ behavior (psychometric variables and creatine kinase levels)

**Fig. 8:**
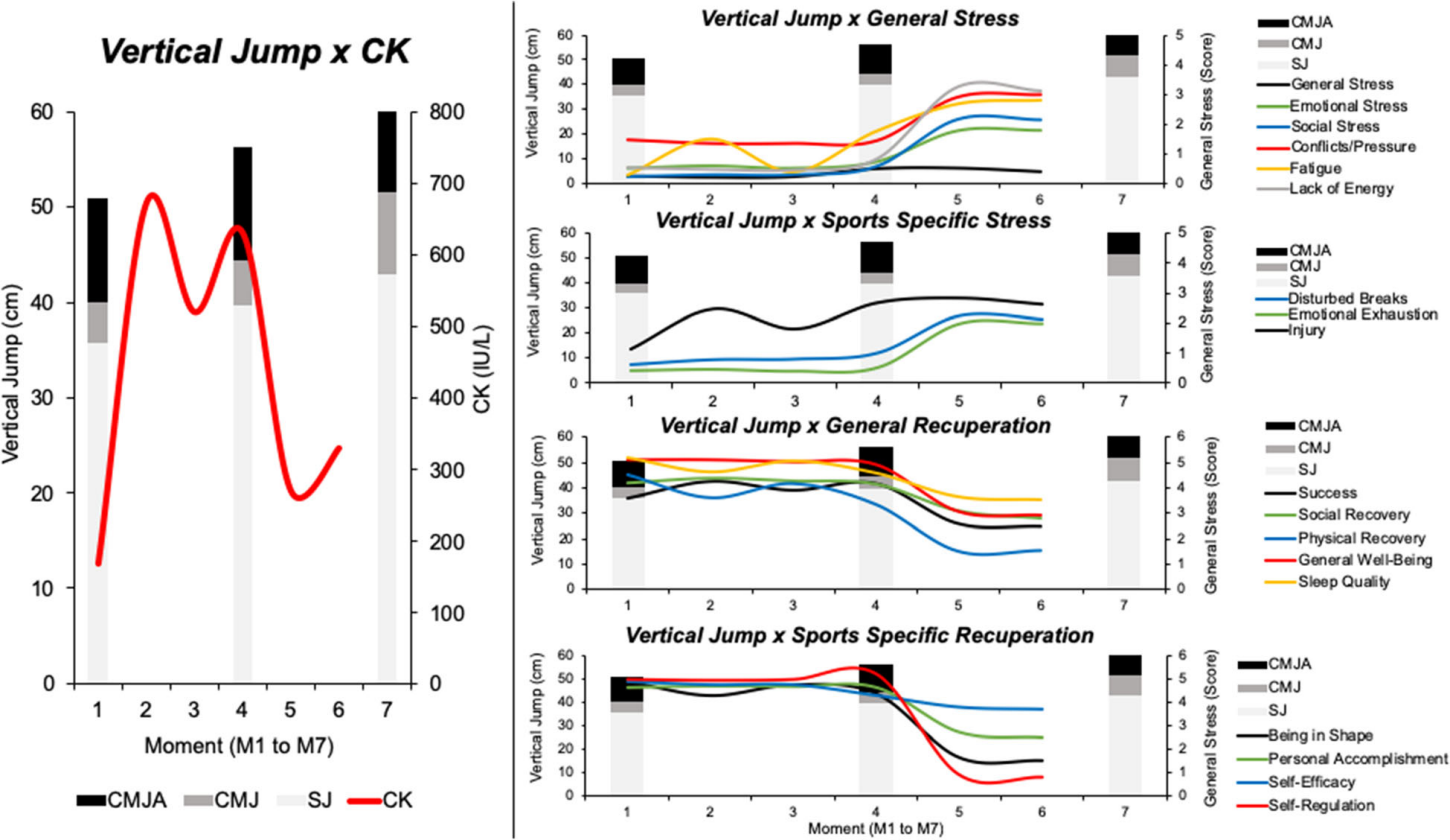
General view of the variables’ behavior (psychometric variables, vertical jumps, and creatine kinase levels)

A general and combined view of the variables analyzed in the present study can be seen in Figs. 7 and 8.

Additionally, secondary analyses were performed, aiming to verify whether there are associations between the stress and recovery perceptions, creatine kinase levels, and the performance parameters. These results demonstrated significant correlations at M1 between general stress and CK levels (*ρ* = 0.582; *p* = 0.047), stress related to conflicts/pressure and CK levels (*r* = 0.655; *p* = 0.021), sport-specific stress related to injury and SJ (*ρ* = − 0.707; *p* = 0.010), sport-specific stress related to injury and CMJA (*ρ* = − 0.631; *p* = 0.028), sport-specific stress related to disturbed breaks and CMJ (*ρ* = -0.599; *p* = 0.039). At M2, a correlation between emotional stress and CK levels was found (*r* = 0.595; *p* = 0.041). Results at M3 showed correlations between general stress and CK levels (*r* = 0.657; *p* = 0.020) and between sport-specific stress related to injury and CK levels (*ρ* = 0.659; *p* = 0.020). Finally, the analyses at M4 demonstrated correlations between general recuperation related to physical recovery and CK levels (*ρ* = 0.583; *p* = 0.037), and sport-specific recuperation related to being in shape and CK levels (*ρ* = 0.683; *p* = 0.010). No significant correlations were found at M5 and M6.

## Discussion

The present study analyzed the stress perception, recovery perception, creatine kinase levels, and performance in jump tests of volleyball athletes during different periods of training during the preseason of a national championship. Our results suggest that in general the perception of both stress and recovery tends to remain stable during the preparatory phase. However, the perception of stress increased and that of recovery decreased in the pre-competitive phase. In addition, CK levels increased soon after the first few weeks of training and tended to remain stable until the pre-competitive phase began, at which time they significantly decreased. As expected, the heights of the jumps increased regularly throughout the whole preseason, regardless of jump type.

Specifically, the emotional stress, social stress, conflicts/pressure, and emotional exhaustion scores remained unchanged until the beginning of the pre-competitive phase (M5 and M6), when they then showed considerable increases. Simultaneously, there were significant decreases in the perceptions of success, social recovery, general well-being, being in shape, personal accomplishment, and self-regulation at M5 and M6 relative to the previous phases of the preparatory period. This behavior can be explained by the high overload of tactical training, by competing in games, and by increases in anxiety preceding the competitions.

In contrast, the fatigue scores, the lack of energy, disturbed breaks, and injury showed early increases in the preseason manifesting before the pre-competitive stage. Similarly, physical recovery, sleep quality, and self-efficacy reduced early. These findings were expected given the progressive increases in training loads peaking at M3 and M4.

In the same way, Horta et al. [19] demonstrated increases in social stress and injury as well as a reduction in the perception of success and general well-being after a short preparatory period in Brazilian elite male volleyball players. However, we obtained stress indices in more scales than Horta et al. [19], which may have been influenced by the duration of the preparatory period and by the number of games in this period. Our study followed the athletes for 11 weeks, with 13 games in the last 9 weeks. On the other hand, Horta et al. [19] accompanied their athletes for only 6 weeks, with only two games in this period. Also, with a follow-up period of 6 weeks, Gonzalez-Boto et al. [18] observed an increase in the stress scales for injury and emotional exhaustion and a decrease in physical recovery, success, being in shape, and self-efficacy in swimmers under preparation for competition.

Different results were found by Purge et al. [17], who did not observe changes in the stress and recovery indices (RESTQ-Index) of elite rowers during a period of 24 weeks of training. The fact that these athletes were younger (mean age 20 years old) than those in the present study (mean age 24 years old) and in Horta et al. [19] (mean age 26 years old) and the lack of participation in competitive games in this period are factors that might have influenced the low levels of stress and high recovery rates observed [17].

Interestingly, in agreement with the findings of the present study, increases in the stress scales and reduction of perceived recovery when the training reached its peak load were observed in other studies [18, 19]. These findings suggest that the RESTQ-Sport scale is sensitive to changes in training loads and can be used in the chronic monitoring of athletes in championship-preparatory phases.

For years, serum CK has been measured and evaluated in exercise science as an essential parameter for the determination of muscular damage. High levels of CK in healthy athletes may be correlated with physical training workloads [14]. In this sense, the responses of CK levels to the different phases of the training periodization during the preseason in our study conformed to expectations.

According to Mougios [27], the ideal range of CK concentrations should vary from 82.00 U/L to 1083.00 U/L for male athletes, and the highest mean value obtained in the different evaluation periods by the athletes investigated in this study was 678.92 U/L (reached M2). On the other hand, Hartmann and Mester [28] consider a more restricted range of values as usual for male athletes, between 200.00 and 250.00 U/L. In the present study, only at the moment of athlete’s presentation (M1, beginning of the preseason) were CK levels close to these values. In all the other periods, values higher than those suggested were observed. It is possible that the standard of the sample of the present study has a chronically high level of CK, given that the same authors report that athletes with these characteristics present high variability and may have acceptable values around 400.00 U/L.

In our longitudinal analysis, CK concentrations showed changes during the preseason. Concentrations increased after the first weeks of training and remained stable until the beginning of the pre-competitive period, at which time they dropped significantly. Freitas et al. [1], in their study of Brazilian volleyball athletes undergoing intensified training loads, observed similar results, even with a shorter follow-up period (25 days). Similarly, but in the longer term, after an initial increment (at 4 weeks) in CK levels, maintenance (for 16 weeks) and a decrease in the last 4 weeks of elite rower training for a championship were observed [17].

The initial increase in CK concentrations observed in the first weeks of training may be justified by the resumption of training after prolonged periods of rest (or detraining), with the neuromuscular system adapting to the stimuli offered by the consecutive exercise sessions. These stimuli are responsible for the muscle damage evidenced by increased CK concentrations, which rise with sarcomeric damage. In fact, according to Brancaccio et al. [14], that higher increment in CK levels is to be expected in individuals with lower physical fitness, as during initial training periods.

The maintenance of CK levels observed during intermediate periods of training, even with increases in workloads along the periodization, has been observed in several studies of elite athletes [1, 17]. This phenomenon can be explained by already structured muscular adaptations and increased tolerance to high training loads. Brancaccio et al. [14] suggested that athletes have higher resting CK levels than untrained subjects, probably because of their greater muscle mass and increased daily training. These factors may contribute to the maintenance found in the cited studies and our findings.

The decrease in CK levels in the final stages of the preseason observed in the present study was also verified in young rowers in preparation for a world championship [8]. Even with an increase in training loads, values after 3 weeks reached one third of those presented at the baseline. In the present study, the training volume in the pre-competitive period was reduced relative to the previous period, which might have contributed to the decreases in the level of this enzyme.

Although some studies suggest that CK levels vary considerably between individuals, a reduction in this variable has been observed in general, especially during periods of training load reduction, after periods of previous high loads [14, 28]. On the other hand, Bachero-Mena et al. [29] did not find significant changes in CK during a complete season in high-level athletes. In the Guilhem et al. [30] study, a significant increase in CK activity occurred in athletes from the preparation to the pre-competitive period, which could be due to higher intensity and specialized exercises during this period. Furthermore, even with weekly training loads higher than those applied in M2, CK values remained significantly lower in the pre-competitive phase. These findings are attributed to muscle adaptation to training, which results in increased tolerance to high loads without significant muscle damage.

The vertical jump in volleyball plays a role in such major actions as serving, spiking, and blocking, and the maximum height reached relates to the effectiveness of scoring during the game [31]. Regarding the performance in the jump tests of the athletes of the present study, a significant increase was observed in the jump heights reached during the preseason, regardless of the type of jump. The values presented at M1 were lower than at the other moments when they were measured (M4 and M7), demonstrating that the training process afforded a positive adaptation in terms of performance. These results are similar to those of Häkkinen et al. [21], who found increases in maximal vertical jumping heights in the SJ (from 30.30 ± 1.70 to 31.60 ± 1.30 cm) and in the CMJ (from 32.80 ± 1.60 to 34.30 ± 1.30 cm) of Finnish women volleyball players in a pre-competitive period of 11 weeks. McGown et al. [20] found a significant improvement in vertical spike jump (from 83.57 ± 5.7 to 93.63 ± 6.1 cm) near the competition. The players tested belonged to the American Olympic team in Los Angeles in 1984. These results, achieved over 2 years and 4 months, are in line with our athletes’ improvements from M1 to M7 regardless of the type of jump during only 16 weeks of preparatory and pre-competitive period.

The secondary analyses of the correlations between the responses to the RESTQ-Sport questionnaire, the CK levels, and the jumping performance during the preseason show interesting results, should be discussed. We believe that the correlations between CK concentrations and general stress and the conflict marker related to conflict and pressure, at moment 1, occurred due to the fact that part of the athletes had little time between the end of the previous season and the beginning of the new training season. The sample of the present study was composed in the largest proportion by athletes who participated in their national teams. Thus, possibly, these athletes had high levels of CK, general stress, and the stress marker related to conflict and pressure, as they were close to the end of the training cycle. Jumping performance is also correlated (negatively), at moment 1, with psychometric variables, in this case, in areas such as interval disturbances and injuries. A lower ability to jump must have occurred in athletes who were not satisfied with the rest intervals and reported having an injury or if they felt likely to be injured. These results indicate that the quality of the interval, the risk of injury, and the ability to jump converge on the same path, which must be taken care of to maintain performance. Due to the fact that these correlations do not occur at other moments, it is believed that uncontrolled demands, prior to the athletes’ presentation, may have influenced the results, considering the athletes of the national teams were not fully recovered.

The correlations between the jumps and the answers to the questionnaire no longer appeared during the preseason (in the other analyzed moments). It is possible that this occurs due to the greater homogeneity in physical conditioning resulting from standardized training among athletes, since they were with similar training loads for being part of the same team. At moments 2, 3, and 4 the correlations between CK concentrations and psychometric variables remain, but there are changes in the set of responses related to the physiological marker. At time 2, CK levels were correlated with emotional stress, and at time 3 the correlations occurred between general stress- and injury-related stress. The increase in loads evidenced by the increase in CK levels seem to interfere in psychological aspects and markers suggestive of injury risk in different ways throughout the season, this behavior being probably explained by the different training demands and the proximity to the competitive period. At time 4, the athletes who reported higher values of physical recovery and being in shape also had higher CK values. This result goes against the researchers’ initial thinking. However, we suggest that the high capacity to support high loads, at this time of the periodization, provided athletes with greater perceptions of physical recovery and being in shape to perform the proposed activities at an intensity closer to their maximum capacity, resulting in higher values of CK. The fact that there is no correlation at other times (M5 and M6), demonstrates that the demands for physical training, such as the absence of strength training, for example, no longer interfere so much in the aspects evaluated in the psychometric variables, being suggested that the variables related to the period of games (competition) affect athletes more than physical training. Thus, these results are interesting for the control of the training load for volleyball athletes, since a simple tool such as the RESTQ-Sport questionnaire can reflect the athletes’ physiological status according to some markers in the preparatory period.

The study has limitations that need to be addressed. The analysis of a single physiological variable of a biochemical nature (CK levels) with high variability could be considered a confounding factor. Therefore, we suggest future studies examine complementary physiological outcomes, such as a biochemical analysis of cortisol/testosterone relationship, rated perceived exertion, and the muscle soreness scale, already mentioned in previous studies [32]. On the other hand, this study presents some strengths, such as (i) the long follow-up duration of high-level volleyball athletes in a preseason period of 16 weeks before a championship and (ii) the integrative approach applied, which analyzed the physiological, psychological, and performance parameters in the same athletes and team. So our study brings some novelties and interesting results like the support to the relationship between a biochemical marker (CK levels), perceived stress and recuperation and performance parameters (vertical jumps), through the analysis of correlations.

## Conclusion

Our results allow us to conclude that in general the perception of both stress and recovery tended to remain stable during the preparatory period. However, the perception of stress increased and that of recovery decreased in the pre-competitive period. Moreover, CK levels increased soon after the first few weeks of training and tended to remain stable until the pre-competitive phase began, at which time the levels significantly decreased. As expected, the heights of the jumps increased regularly during the whole preseason, regardless of the jump type. Thus, although the variables used in training monitoring indicated a higher level of stress and muscle damage, in addition to lower perceptions of recovery during preparation, positive adaptations to the training stimuli were obtained, with resulting improvements in athletes’ performance. Finally, our results showed that the adaptations on stress, fatigue, and performance were not strictly linear in high-level, international volleyball players. Although the alterations follow an expected course, the lines of change have not converged on exactly the same endpoint from different stages of the preseason.

## Data Availability

The datasets used and/or analyzed during the current study are available from the corresponding author on reasonable request. Please contact the authors for data requests.

## Abbreviations

CK: Creatine kinase enzyme
SJ: Squat jump
CMJ: Countermovement jump
CMJA: Countermovement jump arm
M1: First moment of evaluation
M2: Second moment of evaluation
M3: Third moment of evaluation
M4: Fourth moment of evaluation
M5: Fifth moment of evaluation
M6: Sixth moment of evaluation
M7: Seventh moment of evaluation
RESTQ-Sport: Recovery-Stress Questionnaire for Athletes
RESTQ-Index: Stress and recovery indices
